# Conventional and non-conventional antigen-binding sites promote the development and function of chronic lymphocytic leukemia stereotyped subset #4 clones

**DOI:** 10.3389/fimmu.2025.1607189

**Published:** 2025-08-21

**Authors:** Yun Liu, Dzmitry Padhorny, Rosa Catera, Antonella Nicolo, Xiao-Jie Yan, Stan Xiaogang Li, Anastasia Iatrou, Andrea N. Mazzarello, Noemi Destefani, Steven L. Allen, Jonathan E. Kolitz, Kanti R. Rai, Massimo Degano, Paolo P. Ghia, Charles C. Chu, Florian Krammer, Hassan Jumaa, Kostas Stamatopoulos, Dima Kozakov, Nicholas Chiorazzi

**Affiliations:** ^1^ Northwell, New Hyde Park, NY, United States; ^2^ Institute of Molecular Medicine, The Feinstein Institutes for Medical Research, Manhasset, NY, United States; ^3^ Department of Applied Mathematics and Statistics, Stony Brook University, Stony Brook, NY, United States; ^4^ Laufer Center for Physical and Quantitative Biology, Stony Brook University, Stony Brook, NY, United States; ^5^ Institute of Immunology, Ulm University Medical Center, Ulm, Germany; ^6^ Institute of Applied Biosciences, Centre for Research and Technology Hellas, Thessaloniki, Greece; ^7^ Department of Biochemistry, Università Vita-Salute San Raffaele, Milano, Italy; ^8^ Division of Immunology, Transplantation, and Infectious Diseases, IRCCS Scientific Institute San Raffaele, Milano, Italy; ^9^ Medical School, Università Vita-Salute San Raffaele, Milano, Italy; ^10^ B Cell Neoplasia Unit and Strategic Research Program on CLL, IRCCS Ospedale San Raffaele, Milano, Italy; ^11^ Department of Microbiology, Icahn School of Medicine at Mount Sinai, New York, NY, United States; ^12^ Ignaz Semmelweis Institute, Interuniversity Institute for Infection Research, Medical University of Vienna, Vienna, Austria

**Keywords:** chronic lymphocytic leukemia, B cell receptor, antigen, antigen binding, autoreactivity

## Abstract

Immunoglobulins (IGs) made by chronic lymphocytic leukemia (CLL) B cells are unique in that they bind themselves (homo-dimerize). This interaction leads to signal transduction with functional consequences that depend on the affinity of homo-dimerization. We have studied the antigen-binding properties of the IGs from a subset of patients with CLL (Subset #4) that homo-dimerize at high affinity. Previously, we had found that subset #4 IGs bound viable lymphocytes. Our new studies, probing an array of >8,000 antigens, indicate that these IGs also bind influenza virus. Because of the IGs high-affinity homo-dimerization, we asked if the defined foreign- and self-antigenic interactions were mediated by conventional B-cell receptor (BCR) domains or a non-conventional receptor created by homo-dimerization. The studies indicated the latter since abrogation of homo-dimerization eliminated binding to influenza virus and its hemagglutinin and to viable lymphocytes. Using these findings, we modeled a developmental path whereby a naive IgM^+^ B cell with subset #4 heavy and light chain variable domains used the conventional BCR to interact with auto- and foreign antigens and acquire homo-dimerization capacity to create the non-conventional antigen-receptor when transitioning to a leukemic cell. Future studies will determine if this process is an idiosyncratic occurrence or a physiologic principle.

## 1 Introduction

Signaling through the B-cell receptor (BCR) for antigen is critical for the development and maturation of normal and neoplastic B lymphocytes in certain B-cell lymphoproliferative disorders (1, 2). Signaling occurs when the clonally restricted surface membrane immunoglobulin molecule (smIG) on a B cell interacts with antigens that are extrinsic to the smIG (3–5) (classical BCR signaling) or are intrinsic to the smIG (6–9) (autonomous BCR signaling). B lymphocytes experience both types of signaling. Classical BCR signaling is required for triaging autoreactivity for developing and mature B cells (10–12), leading to survival, expansion, and maturation upon reaching maturity (13, 14). Autonomous signaling occurs mainly early in development at the pre-B cell stage (6–8), allowing an emerging B lymphocyte to move along the maturational pathway without interacting with the external microenvironment. Nevertheless, a fraction of mature normal B lymphocytes must receive signals autonomously since such B cells can expand and become transformed in patients with chronic lymphocytic leukemia (CLL) and some other allied conditions (9, 15). In CLL, autonomous signaling comes about by the smIG on a CLL clone homodimerizing with an identical partner on the B-cell surface (9).

The occurrence of IG-IG interactions of identical recombinant CLL IGs has been shown in antigen-binding assays (16) and physically demonstrated by X-ray crystallography (17). B cells bearing CLL IGs that self-associate spontaneously induce Ca^++^ flux as a measure of cell action. The affinity of homodimerization varies among CLL IgGs (17), and hence the functional consequences of cell activation to B cells bearing self-associating IGs can vary.

CLL IGs also exhibit unique structural features (18), such as the over abundant use of certain IG heavy chain variable (IGHV) genes compared to the genes expressed in healthy people (19), and the apparent selection for IGHVs that associate with identical IG heavy chain diversity (IGHD) and IG heavy chain joining (IGHJ) genes in people with the disease (20–23), and are referred to as “stereotyped BCRs”. Some of these IGHV-IGHD-IGHJ (IGHV-D-J) rearrangements are also paired with similar IG light chain kappa or lambda variable (IGK/LV) and IG light chain kappa or lambda joining (IGK/LJ) gene rearrangements (IGK/LV- IGK/LJ). Notably, the occurrence of stereotyped BCRs is surprisingly common among patients with CLL, approaching 41% of patients (24). Also remarkable is finding identical or chemically similar somatic mutations at the same position in the IGHV from multiple CLL clones in the same stereotyped BCR (“stereotyped mutations” (25)). Finally, patients with clones bearing the same stereotyped BCRs often have very similar clinical courses, and their leukemic B-cell clones can carry similar genomic abnormalities (26, 27).

Here, we have studied a subset of CLL patients with a stereotyped BCR that exhibits many of the above features, patients who fall into stereotyped subset #4 (SS#4). These patients bear CLL clones that exhibit an IGHV-D-J rearrangement comprised of IGHV4-34, IGHD5-18, and IGHJ6 that is associated with IGKV2-30 (28). Notably, the IGHV-D-J of patients in subset #4 can display stereotyped mutations in the IGHV4–34 and in the IGKV2-30 (29), suggesting exertion of selective pressure to select B-cell clones bearing these amino acids during maturation (25, 30). Moreover, the leukemic cells in these patients always use the IgG constant region (31). Clinically, these patients are remarkable in having a more favorable clinical course compared to that of other patients (27, 32), despite often developing the disease at a younger age. *In vitro*, unmanipulated leukemic B cells from patients in SS#4 fail to respond to BCR engagement by surrogate antigen (anti-IG) (33, 34), suggesting that these clones are functionally anergic *in vivo*. Moreover, recombinant SS#4IgGs (SS#4IgGs) homo-dimerize with high avidity (17), possibly explaining their anergic state and the relatively benign clinical courses of patients with these clones. Finally, SS#4IgGs differ in antigen reactivity from most other CLL IGs, in that they do not bind autoantigens (e.g., DNA, IgG, myosin) or apoptotic cells (28, 35–37) with which most other CLL IGs do. Rather, they react with viable lymphoid cells (36, 38) and this reactivity requires a stereotyped mutation at the junction of the IGKV and IGKJ rearrangement (38).

Because of this unusual BCR binding pattern, we asked if SS#4IgGs could react with foreign antigens, screening a panel of >8,000 candidates. This revealed selective binding to influenza virus, and its hemagglutinin. Strikingly, this binding only occurred when the variable domains of the SS#4IGs were associated with the IgG isotype and in the homodimerized state. Molecular modeling ascribed a broad receptor interface that did not involve VH CDR3 but mainly involve framework regions and constant regions of the self-associated SS#4IgG. Thus, avid homodimerization, which in itself leads to BCR signaling, also allowed binding to the same BCR via a non-conventional hemagglutinin binding site created by self-association. These features allowed tracking a possible development path whereby a virgin, normal B lymphocyte expressing an unmutated SS#4IgM BCR transitioned to a leukemic SS#4IgG leukemic clone by focusing on the influences of the conventional BCR binding site and the non-conventional binding.

## 2 Materials and methods

### 2.1 Study approval

All sample collections and studies performed were approved by the Institutional Review Board of The Feinstein Institutes for Medical Research in accordance with the Declaration of Helsinki.

### 2.2 Samples from patients with CLL

Blood was obtained from patients after obtaining written informed consent. The sex distribution among patients with CLL is ~2:1, male to female, and collection of samples was not influenced by sex. The samples collected and used represent the male to female ratio seen in the disease.

### 2.3 Cloning, expression, and purification of CLL mAbs

RNA from blood mononuclear cells of CLL patients was extracted and converted into cDNA as described (19). Cloning, expression, and purification of mAbs were performed as reported (62).

### 2.4 Site-directed mutagenesis

Targeted mutagenesis of the IGHV-IGHD-IGHJ and IGKV-IGKJ DNA sequences of CLL IGs was performed by GENEWIZ, Inc. (South Plainfield, NJ).

### 2.5 Antibody specificity profiling

Purified CLL IGs were sent to the vendor (Invitrogen) to probe ProtoArray^®^ Human Protein Microarray V 4.0. Details of the assay are available in the **Supplementary Material** section.

### 2.6 IG binding to influenza A viruses or purified hemagglutinins measured by enzyme-linked immunosorbent assay

Influenza virus A/TEXAS/1/77/H3N2 was purchased from BiosPacific, CA, USA. Recombinant hemagglutinins were purified as described (63). Viruses or hemagglutinins were coated at 2μg/ml to Polystyrene plates (Nunc, Roskilde, Denmark) at RT for 30min. Plate was saturated with blocking buffer consisting of phosphate-buffered saline (PBS; pH 7.4; Gibco) supplemented with 0.1% Tween 20 (PBS-T), 3% human serum albumin (Calbiochem, CA, USA) and incubated at RT for 2h. IGs were diluted in PBS and incubated on plates at 4°C for overnight. The plates were then washed 3 times with PBS-T and bound IGs were detected with horseradish peroxidase conjugated goat anti-human IgG. After 1h, plates were washed 4 times with PBS-T and developed for 15min with TMP Sure Blue 1-component substrate (KPL, Gaithersburg, MD), stopped with 1M HCl, and absorbance measured at 450nm. HAs from groups 1 and 2 from 6 H3N2 strains (A/Texas/1/77, A/Hong Kong/1/68, A/Alabama/1/8, A/Philippines/2/82/, A/Panama/2007/1999, A/Wyoming/3/03), 4 H1N1 strains (A/Fort Monmouth/1/47, A/Texas/36/91, A/New Caledonia/20/99, A/California/04/09), and one H2N2 strain (A/Japan/305/57) were available and used.

### 2.7 IG binding to DNA by ELISA

To determine IG binding to double (ds) and single stranded (ss) DNA, the precoated ELISA and reagents of QUANTA Lite™ dsDNA and QUANTA Lite™ ssDNA ELISA kits (INOVA Diagnostics, Inc., San Diego, CA) were used. Calibrators were not used in this ELISA as signal to unit conversion was not required. IGs were tested following the manufacturer’s instructions at indicated concentrations. The signals were used for plotting.

### 2.8 Cell immunofluorescence and flow cytometric analysis

The human Ramos B cell line cells were induced to undergo apoptosis by γ-irradiation (4000-5000R) ~15h before staining. Cells were then incubated with CLL IGs (5 - 50 μg/mL) for 1h at 4°C and binding detected by FITC-conjugated F(ab’)_2_ goat anti-human IgG (Southern Biotech, Birmingham, AL). Apoptosis was measured by staining with Annexin V-PE (BD Pharmingen, BD Biosciences, San Jose, CA) as recommended by the vendor. Samples were acquired using a LSRFortessa flow cytometer (Becton Dickinson, San Jose, CA) and analyzed using the FlowJo (LiveTree, San Diego, CA) software.

### 2.9 Autonomous cell signaling measured by Ca^++^ flux

The ability of SS#4IGs to mediate signal transduction was tested using the TKO cell line (7) that does not express pre-B or mature B-cell IG components due to inactivation of the RAG2 and γ5 genes. The cell is also functionally impaired by making the adaptor molecule, SLP65, which is essential in BCR signal transduction, unavailable until exposed to 4-hydroxytamoxifen (4-OHT). The approaches used for inserting SS#4IGs into TKO cells and the subsequent measurement of Ca^++^ flux are as reported (9, 64). Details of these are available in **Supplementary Material**.

### 2.10 Molecular modeling of the interaction of SS#4IgG with influenza virus hemagglutinin

The protein structures of CLL240 asymmetric antibody dimers (PDB ID: 5DRX) and hemagglutinin of the A/Hong Kong/1/1968 (H3N2) influenza virus (PDB ID: 5T6N) were docked using the ClusPro server (57). Due to the symmetrical nature of hemagglutinin’s homo-trimeric biological assembly, repulsive masking was applied to one monomer out of three to eliminate symmetrically identical protein-protein complexes resulting from docking. After masking was applied to the structure, the three step docking process was initiated: rigid body docking, clustering of 1000 lowest energy structures, and relaxation of the resulting structures using energy minimization. Rigid body docking was performed using PIPER, an algorithm based on Fast Fourier Transform (FFT). The 1000 lowest energy poses were then taken, and clusters were formed for neighboring poses with <9 Angstrom Root-Mean-Square Deviation (RMSD). The representative poses from each cluster were then relaxed via energy minimization and ranked based on cluster size and energy. Next, the top-ranking docking solution which involves both antibody monomers interacting with the hemagglutinin (as suggested by experimental data) was selected as the final model. The resulting model was analyzed and amino acids within 5 Angstroms of each other were defined as residues forming the protein-protein interface. Modeling can be viewed at: https://figshare.com/articles/figure/Structure/28239857?file=51805436.

### 2.11 Influenza virus cytopathic inhibition assay

Virus A/Philippines/2/1982/H3N2 was grown in 8- to 10-day-old embryonated chicken eggs (Charles River Laboratories) at 37°C for 48h. MDCK cells were seeded at a concentration of 2×10^5^ cells/ml were seeded in white polystyrene 96-well plates (Costar Corning). The next day, 60µl of mAbs serially diluted 2-fold starting from 400μg/ml in RPMI 1640 medium (Life Technologies) was incubated with 60µl of virus dilution (1,250 PFU/60µl) for 1h at RT on a shaker. MDCK cells were washed once with 220µl of PBS, and then 100µl of the virus-serum mixture was added to MDCK cells. The mAb-virus mixture and the MDCK cells were incubated overnight at 37°C. For phase contrast microscopy, a poly-D-lysine treated coverslip placed in well was used for MDCK cell growth and neutralization. The coverslip was gently washed with PBS and host cell morphology was captured.

### 2.12 Statistics

For statistical analysis, GraphPad Prism software was used. The statistical significance (*P*-value) of two group comparison was calculated using unpaired student T-test. Statistical significance was defined by a *P*-value of less than 0.05 (2-tailed). Data are presented as mean ± SEM, unless otherwise indicated.

### 2.13 Data availability

Data produced will be shared to ensure transparency and reproducibility and to avoid duplication of research effort and expense. Flow cytometry files will be shared in.fcs format. All processed ELISA data will be made available in.xls format. Modeling data can be accessed at https://figshare.com/articles/figure/Structure/28239857?file=51805436.

## 3 Results

### 3.1 CLL subset 4 IGs bind influenza A viruses and hemagglutinins from groups 1 and 2

Most CLL IGs are polyreactive (39–45), binding a variety of microbes (35, 46–48) and diverse sets of (auto)antigens, including apoptotic cells (35–37, 49). Contrary to this paradigm, IGs from SS#4 clones react with an unidentified surface membrane molecule on viable human lymphocytes (38).

To determine if SS#4IGs bound to other, unappreciated targets, we probed ProtoArray^®^ Human Protein Microarrays v4.0, a platform containing 8,222 antigenic targets, with three SS#4IGs (183, 240, 342) and 25 other CLL patient derived rIGs (**Supplementary Table S1**). Among the latter, 16 belonged to other stereotyped BCR subsets and 9 were not assigned to a subset.

**Figure 1:**
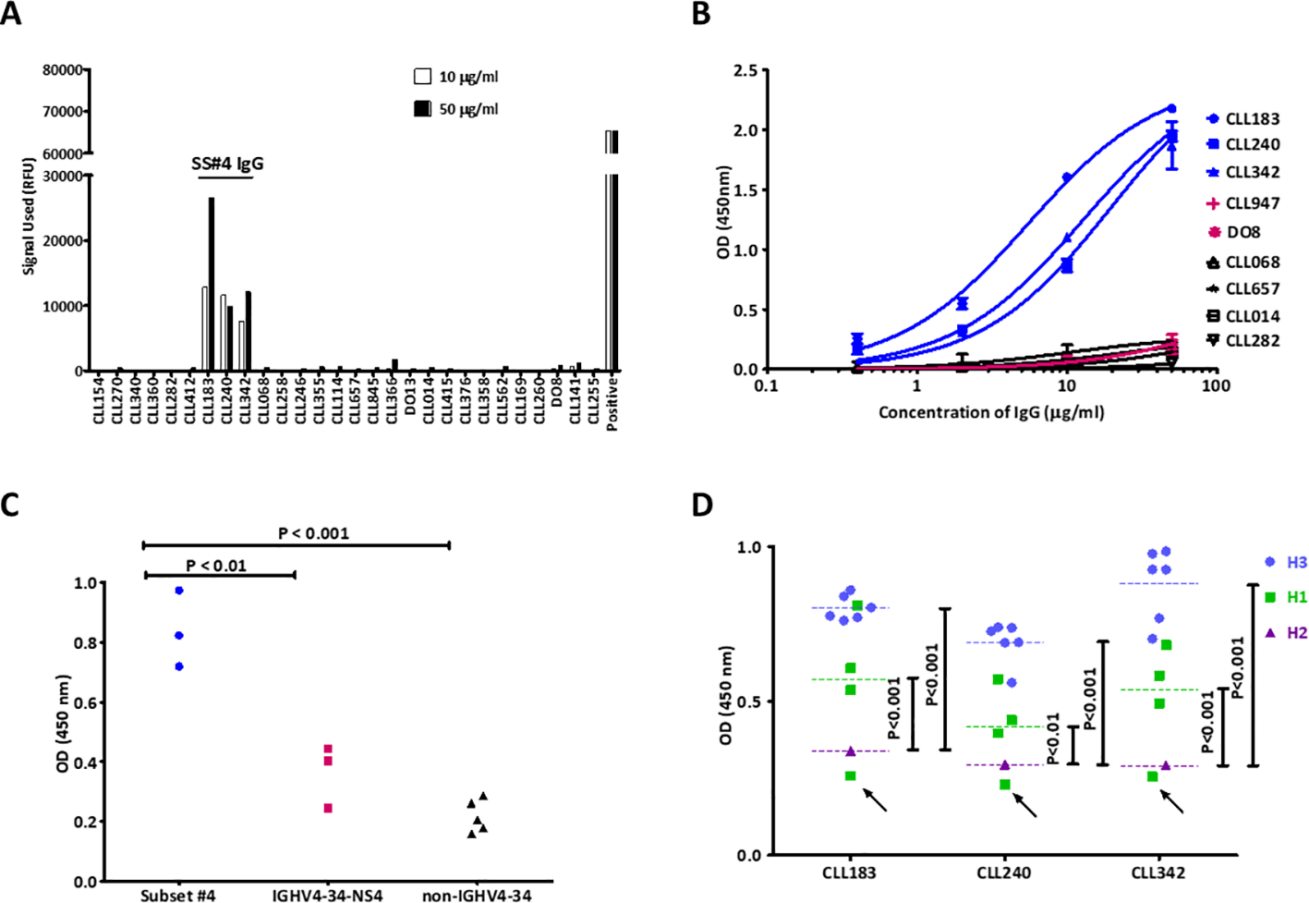
SS#4IgGs bind influenza A viruses and hemagglutinins. **(A)** Protoarray^®^ Human Protein Microarray screening. Three SS#4IgGs (183, 240, 342) and 25 non-SS#4IgGs were tested. Bars are the average of the two measurements (10μg/ml, white, and 50μg/ml, black). Calmodulin kinase Iiα probed with a murine kinase specific mAb was used as positive control. IG ID identifies the CLL patient from whom mAb was derived (**Supplementary Table S1**). **(B)** Measurement of SS#4IgG binding to influenza virus A/Texas/1/77 (H3N2) by ELISA. SS#4IgGs (CLL183, 240, 342) and IGs from CLL stereotyped subsets #2 (CLL282, IGHV3-21), #6 (CLL 068, IGHV1-69), #8 (CLL657, IGHV4-39) and #31 (CLL014, IGHV1-69) and CLL-derived IGHV4-34-NS4 IGs (CLL947, DO8) were analyzed. Blue: SS#4IgGs; Red: IGHV4-34-NS IgGs; Black: non-IGHV4-34-expressing IgGs. Error bars indicate SEM. **(C)** Measurement of SS#4IgG binding to recombinant H3 of influenza virus A/Texas/1/77 (H3N2) in ELISA. SS#4IgGs (CLL183, 240, 342; Blue dots) and 3 CLL-derived, IGHV4-34-NS4 IgGs (Red squares), and 5 CLL-derived, non-IGHV4-34-expressing IgGs (Black triangles) were tested. **(D)** Measurement of SS#4IgGs binding to groups 1 and 2 HAs from 6 H3N2, 4 H1N1, and 1 H2N2 strain. See Methods for HA strains. Binding to each HA subtype is indicated by symbols and median by dashed line; H3s (Cyan), H1s (Green), and H2 (Purple). H1 from A/California/04/09 is indicated by black arrow.

Each SS#4IgG bound selectively and significantly, in a dose dependent manner, to influenza A/Texas/1/77 virus (H3N2) on the array (**Figure 1A**). In striking comparison, none of the other 25 CLL-derived mAbs did. The non-binders included two IGHV4–34 IGs that do not use IGHD5-18/IGHJ6 (CLL141, DO8). (We will refer to these IGHV4-34, non-subset #4 IGs hereafter as “4-34-nSS#4IGs”). Although antibodies induced during (influenza) viral infections often use IGHV1-69 (50, 51), a series of CLL-derived IGs from clones expressing IGHV1–69 belonging to subsets 6, 7A, 7B, and 31, and an IGHV1–69 not belonging to a subset (CLL358), did not bind the virus (**Figure 1A**).

To confirm this reactivity, we performed an ELISA using H3N2 A/Texas/1/77 virus as the target. Consistent with the array findings, each SS#4IG showed dose-dependent binding that fit one site-specific binding curves (**Figure 1B**). In contrast, two 4-34-nSS#4IGs and IGs from stereotyped subsets 6 and 31 (both IGHV1-69), 8 (IGHV4-39) and 2 (IGHV3-21) did not react with virus, even at the highest concentration tested.

The surface glycoprotein hemagglutinin (HA) of influenza viruses is a major determinant of antigenicity and pathogenicity (52, 53). Therefore, we compared binding of SS#4IGs and other CLL-derived IGs to the HA from A/Texas/1/77 (H3N2). SS#4IGs bound well to HA; this reactivity was significantly greater than that for 4-34-nSS#4IGs or non-IGHV4-34IGs (**Figure 1C**).

Next, we tested SS#4IGs for binding to HAs from a series of viral strains: 6 H3N2 strains from the 1968 A/Hong Kong/1 pandemic (54); 4 H1N1 strains from the 2009 “Swine Flu” pandemic (55), and 1 H2N2 strain from the 1957-1958 “Asian Flu” pandemic (54) (**Figure 1D**). Each SS#4IG bound significantly and similarly to all 6 H3s and to 3 of the 4 H1s, the latter with varying effectiveness. Notably, none of the SS#4IGs reacted with H1 from A/California/04/2009 (H1N1) nor with the H2 of A/Japan/305/57 (H2N2). So, SS#4IGs bind to HA subtypes in this order: H3 > H1 >> H2.

### 3.2 Homo-dimerization of subset #4 IgGs is required for recognition of foreign- and auto-antigens

CLL IGs can interact with themselves (9, 16), a form of autoantigen binding. The crystal structures of SS#4IgGs 183 and 240 in the homodimerized, self-associated state have been solved (17). In the complex formed by self-association of two SS#4IgGs, amino acids in the FR1 and CH1 of one IG molecule form an epitope which acts as an “Antigen”, and this surface embraces the side chains of HCDR3 of the “Receptor” IG. Distinct interactions include a salt bridge between FR1 Glu^17H^ of the Antigen IG and Arg^116H^ in the VH CDR3 of the Receptor IG, an interaction between the side chain of Tyr^117H^ of the Receptor IG and a hydrophobic pocket formed by the Antigen IG, and hydrogen bonds and van der Waals’ interactions between VH CDR3 residues Tyr^118H^, Tyr^119H^, and Tyr^120H^ with the IgG CH1 domain (17). (All amino acid positions listed here follow the ImMunoGeneTics (IMGT) numbering system (56). To compare the IMGT numbering used here with that used in the crystallography studies see **Supplementary Figure S3**).

**Figure 2:**
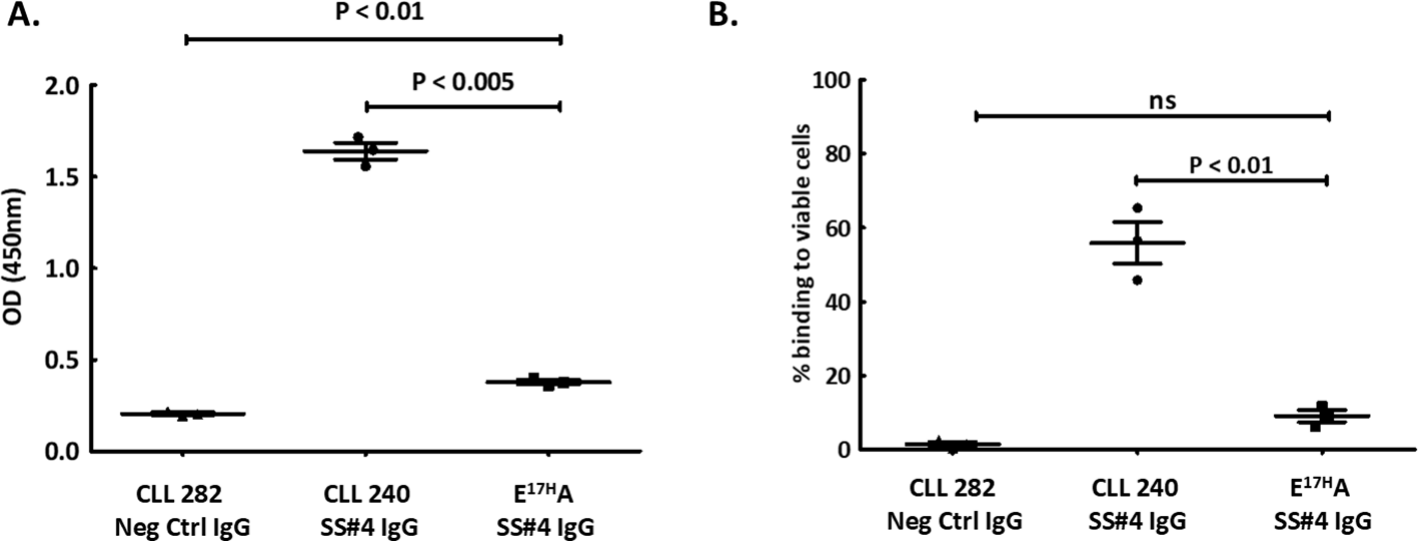
Homo-dimerization of SS#4IgGs is required for foreign and auto-antigen recognition. A non-self-associating mutant of SS#4IgG240, E^17H^A, created by substituting Glu17 in FR1, was tested for binding to influenza virus (foreign) or auto (viable B cell surfaces) antigens and compared to wt SS#4IgG 240 or negative control (CLL IgG 282). **(A)** A/Texas/1/77 (H3N2) influenza viruses. The non-self-associating mutant showed significantly reduced binding compared to wt SS#4IgG 240 (FR1 240E^17H^A vs. wt CLL 240 IgG:0.38 ± 0.022 OD vs. 1.64 ± 0.080 OD, *P*<0.005) while higher than the binding to negative control IgG 282 (0.21 ± 0.012 OD, *P*<0.01); **(B)** Annexin V^-^, viable human B cells (Ramos). Non-self-associating mutant exhibited significantly less binding to human Ramos B cells compared to wt SS#4IgG 240 (FR1 240E^17H^A vs. wt CLL 240 IgG:9.07% ± 2.80% vs 55.88% ± 9.74%, *P*<0.01). This binding was comparable to that of the negative control (1.49% ± 1.58%, ns). Data are from 3 independent experiments. Error bars represent SEM.

Based on these crystallographic findings, we tested if binding to influenza virus was through a standard interaction between HA3 and the CDRs of the SS#4IGs or if binding required a homo-dimerized complex. To address this, we also made use of the finding that replacing Glu17 with Ala (E^17H^A) on VH FR1 leads to loss of self-association (17), presumably because the variant cannot create the SS#4 Antigen IgG that interacts with the VH CDR3 Receptor IgG. When testing the abilities of the non-self-associating variant (FR1 240E^17H^A) and the self-associated wild type (wt) SS#4IgG to react with influenza virus by ELISA (**Figure 2A**), we found that the variant exhibited 77% reduced binding compared to the wt SS#4IgG (*P*<0.005).

Next, we checked if binding to the surface membranes of viable human B lymphocytes also required a dimerized SS#4IgG. Comparing reactivity of the FR1 240E^17H^A variant and the wtSS#4IG with viable B cells by flow cytometry revealed 84% less binding of the non-self-associating variant than the wt SS#4IgG (*P*<0.01) (**Figure 2B**).

Thus, SS#4IgGs interact with a foreign antigen (influenza virus hemagglutinin) and a self-antigen (viable lymphoid cell membranes) only when in the self-associated, homodimerized state.

### 3.3 Molecular modeling of the interaction of influenza virus hemagglutinin with a non-conventional binding site created by homodimerization

**Figure 3:**
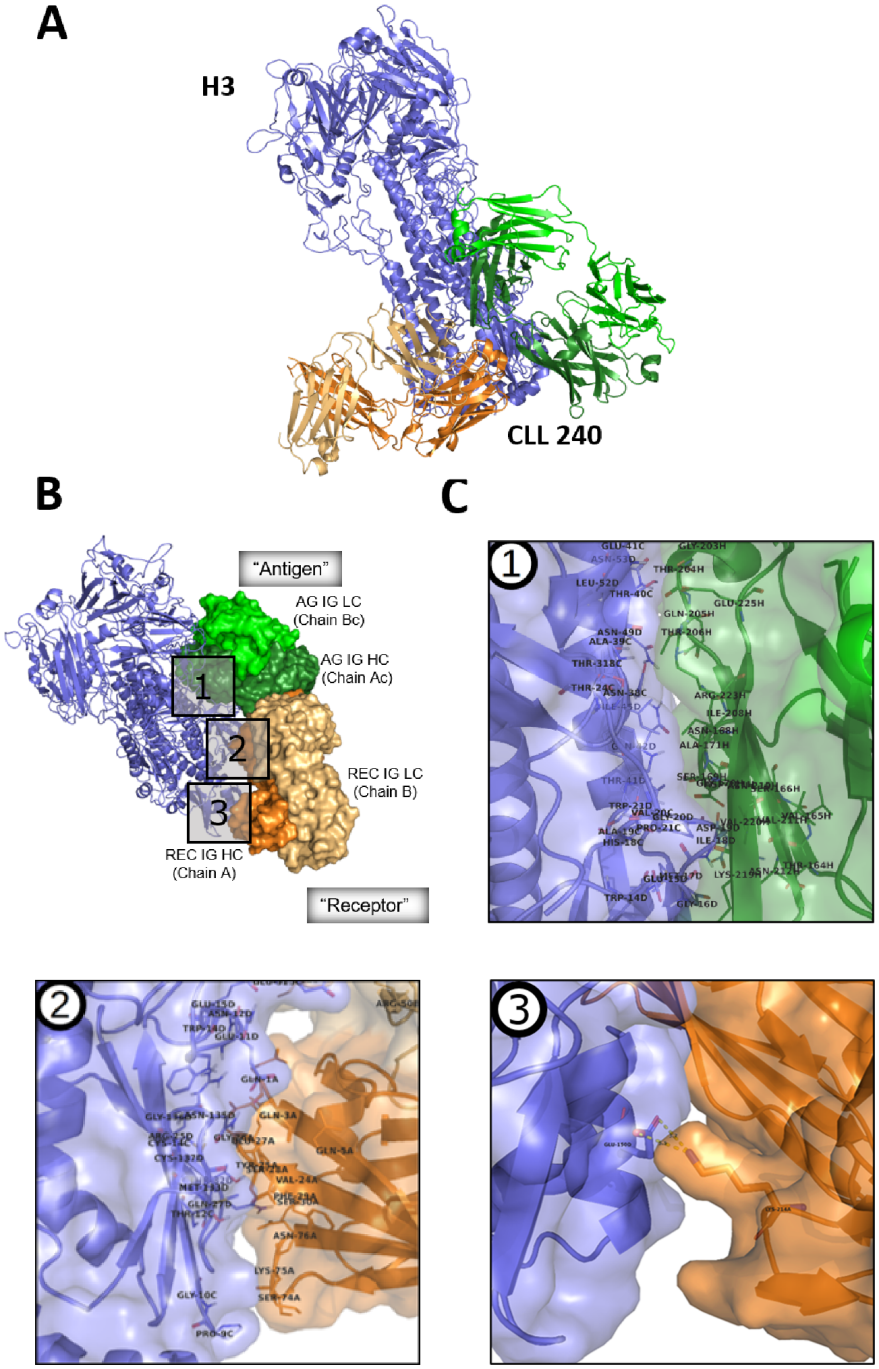
Modeling the interaction between SS#4IgG240 asymmetric dimer and hemagglutinin subtype 3. **(A)** CLL240 asymmetric antibody dimer was docked to the hemagglutinin of A/Hong Kong/1/1968 (H3N2) influenza virus. The top-ranking solution is shown (see Methods). Blue: hemagglutinin; Orange: “Receptor” antibody; Green: “Antigen” antibody. **(B)** Interface of hemagglutinin with asymmetric dimer. Blue: Hemagglutinin; Green: “Antigen” antibody, AG IG LC – Antigen IG Light Chain(Chain Bc, light green), AG IG HC - Antigen IG Heavy Chain (Chain Ac, dark green); Orange: “Receptor” antibody, Rec IG LC – Receptor IG Light Chain (Chain B, light orange), Rec IG HC – Receptor IG Heavy Chain (Chain A, dark orange); **(C)** Major features of the binding interface of HA with (1) “Antigen IG”; (2) “Receptor IG”, and (3) the constant region of the H chain of “Receptor IG”. Residue numbering is that used in crystallization study (17); the corresponding IMGT numbering is in **Supplementary Figure S4A**.

Since homo-dimerization was required for foreign and self-antigen binding, we reasoned that the antigen-binding site of the self-associated complex might not be a conventional one, since at least parts of the variable domains of the component IGs would not be available because of homo-dimerization. This was tested virtually by docking the CLL240 IgG homodimer to the HA of A/Hong Kong/1/1968 (H3N2) influenza virus, using the ClusPro protein docking server (57) (**Figure 3A**).

Using this model, we first tried to determine the structural reasons for the differences in bindings to HA3, HA1, and HA2 (**Supplementary Figure S1**). Many residues within the HA3 stem region that participate in the interaction with the dimerized SS#4IGs are conserved in the HA from strain A/Fort Monmouth/1/47 (H1N1). The residues differing between the H3 and H2 subtypes are highlighted in red (**Supplementary Figure S1**). The differences are most substantial in the interface region, which is consistent with CLL240 binding to HA1 as well as HA3, but not HA2 in ELISA (**Figure 1D**).

Next we focused on understanding the specific modeled interactions between the self-associated SS#4IgG and HA3 (**Figure 3A**). Notably, the homo-dimerized IgG (green and orange) interacts preferentially with the stem region of the HA (slate blue), not the globular head region (dark blue), the latter being more diverse among strains than the former. Three distinct portions of the homodimer are involved (**Figure 3B**): an extensive area of the H chain of the Antigen IG (AG IG HC); a targeted area of the variable domain of the IG H chain of the Receptor IG (REC IG HC), and a single interaction of constant region of H chain of the Receptor IG.

Concentrating on each portion individually (**Figure 3C**) indicates that, for the first portion, there are 23 contact residues in the constant region of the Antigen IG that likely interact with the hemagglutinin (**Figure 3C**, box 1; **Table 1**, columns 1 and 2). Since there are multiple amino acids in the HA with which these could partner, specific interactions are not listed in the Table. (Crystal study numbering is used here and in rest of this section. For corresponding IMGT numbering see **Supplementary Figure S3**). For the second portion, there are 18 amino acids of the Receptor IG that can be assigned specific complimentary residues in the HA; 14 of these are in the variable domain of the H chain and 4 in the variable domain of the L chain. Notably, of the interacting residues in these variable regions, for the H chain 6 are in FR1, 3 in FR3, and 5 are in CDR1; for the L chain, 2 interacting residues are in FR2 and 2 in CDR2 (**Figure 3C**, box 2; **Table 1**, column 3). Thus, 11 interactive residues are in FR and 7 in CDRs. Finally, for the third portion of the homodimerized SS#4IgG that interacts with HA3, there is only one interacting amino acid, which is the Lys at position 214 of the Receptor IG (**Figure 3C**, box 3; **Table 1**, column 3). Note that for homo-dimerization to occur, the Lys at position 214 is required, albeit in the Antigen (not Receptor) IG.

**Table 1:** Residues involved in the interactions of SS#4IgG 240 homo-dimer and HA3.

Hemagglutinin				Antigen IG			Receptor IG		
**HA2 monomer 1 (Chain D)**	**Corresponding interacting area**	**HA2 monomer 2 (Chain F)**	**Corresponding interacting area**	**Heavy chain (Chain Ac)- Ag HC (Chain Ac)**	**AA position on Ig Segment**	**Corresponding interacting area**	**Heavy Chain (Chain A) - Rec HC (Chain A)**	**AA position on IG Segment**	**Corresponding interacting area**
FF150, GLU	Not shown	11, GLU	Box 2	164, THR	Constant region	Box 1	1, GLN	FR1 region	Box 2
153, ARG	Not shown	12, ASN	Box 1	165, VAL	Constant region	Box 1	3, GLN	FR1 region	Box 2
154, ASN	Not shown	14, TRP	Box 1	166, SER	Constant region	Box 1	5, GLN	FR1 region	Box 2
158, ASP	Not shown	15, GLU	Box 1	168, ASN	Constant region	Box 1	7, TRP	FR1 region	Box 2
**HA1 monomer 2 (Chain E)**	**Corresponding interacting area**	16, GLY	Box 1	169, SER	Constant region	Box 1	24, VAL	FR1 region	Box 2
9, PRO	Box 2	17, MET	Box 1	170, GLY	Constant region	Box 1	25, TYR	FR1 region	Box 2
10, GLY	Box 2	19, ASP	Box 1	171, ALA	Constant region	Box 1	26, GLY	CDR1 region	Box 2
12, THR	Box 2	20, GLY	Box 1	203, GLY	Constant region	Box 1	27, GLU	CDR1 region	Box 2
14, CYS	Box 2	21, TRP	Box 1	204, THR	Constant region	Box 1	28, SER	CDR1 region	Box 2
18, HIS	Not shown	25, ARG	Box 2	205, GLN	Constant region	Box 1	29, PHE	CDR1 region	Box 2
19, ALA	Box 1	26, HIS	Box 2	206, THR	Constant region	Box 1	30, SER	CDR1 region	Box 2
20, VAL	Box 1	27, GLN	Box 2	208, ILE	Constant region	Box 1	74, SER	FR3 region	Box 2
21, PRO	Box 1	28, ASN	Box 2	210, ASN	Constant region	Not shown	75, LYS	FR3 region	Box 2
24, THR	Box 1	32, THR	Box 2	211, VAL	Constant region	Box 1	76, ASN	FR3 region	Box 2
38, ASN	Box 1	41, THR	Box 1	212, ASN	Constant region	Box 1	**Light chain (Chain B) - Rec LC (Chain B)**	**AA position on Ig Segment**	**Corresponding interacting area**
39, ALA	Box 1	42, GLN	Box 1	217, ASN	Constant region	Not shown	**44, ARG**	**FR2 region**	**Not shown**
40, THR	Box 1	45, ILE	Box 1	219, LYS	Constant region	Box 1	**50, ARG**	**FR2 region**	**Box 2**
41, GLU	Box 1	49, ASN	Box 1	220, VAL	Constant region	Box 1	**61, SER**	**CDR2 region**	**Not shown**
44, GLN	Not shown	52, LEU	Box 1	221, ASP	Constant region	Not shown	**62, GLY**	**CDR2 region**	**Not shown**
48, THR	Not shown	53, ASN	Not shown	223, ARG	Constant region	Box 1	**Heavy Chain (Chain A) - Rec HC (Chain A)**	**AA position on Ig Segment**	**Corresponding interacting area**
50, LYS	Not shown	56, ILE	Not shown	225, GLU	Constant region	Not shown	214, LYS	Constant region	Box 3
275, ASP	Not shown	133, MET	Not shown	228, SER	Constant region	Not shown			
289, PRO	Not shown	135, ASN	Not shown	**Light chain (Chain Bc) - Ag LC (Chain Bc)**	**AA position on Ig Segment**	**Corresponding interacting area**			
292, LYS	Not shown	137, CYS	Not shown	220, CYS	Constant region	Not shown			
318, THR	Not shown								
325, GLU	Box 1								

The chain, position number, and depicted interacting interfaces are listed. For the residues of the “Antigen IG” and the “Receptor IG”, the position in the IG segment is also indicated. “Not shown” indicates an interactive residue that cannot be shown on the Figure because of the angle of the diagram.

All residues are color coded to match the colors used in **Figure 3B**. Blue: Hemagglutinin; Green: “Antigen”antibody, Antigen IG Light Chain (Chain Bc) - light green, Antigen IG Heavy Chain (Chain Ac) - dark green; Orange: “Receptor” antibody, Receptor IG Light Chain (Chain B) - light orange, Receptor IG Heavy Chain (Chain A) - dark orange.

Thus, the interaction between influenza virus HA3 and the non-conventional binding site is through amino acids in the constant region of the Antigen IG. For the Receptor IG, residues in the constant region and the FR contribute more than the CDRs.

### 3.4 The non-conventional binding site created by homodimerization of SS#4IgGs is relatively stable

The functional capacity of such a non-conventional, conformational binding site depends on its stability and hence the duration that the Antigen-Receptor complex is intact. To test this, we made use of the finding that homo-dimerization of CLL IGs induces autonomous BCR signaling in an experimental system in which IGs are inserted into the membrane of a reporter B cell line (TKO) that only permits BCR signaling when exposed to 4-OHT (8, 9).

Using this system, we determined the ability of soluble, non-associable components of SS#4IgGs or of the IGs in normal human serum to disrupt the homo-dimerized complex and thereby abrogate autonomous signaling. Specifically, we co-transfected TKO cells with constructs containing membrane versions of wt SS#4IgG and Igκ, and then exposed these cells to the soluble, “Antigen-only” variant (R^116H^A/Y^117H^A) or to the “Receptor-only” variant (E^17H^A) of the 3 SS#4IgGs, 183, 240, 342.

**Figure 4:**
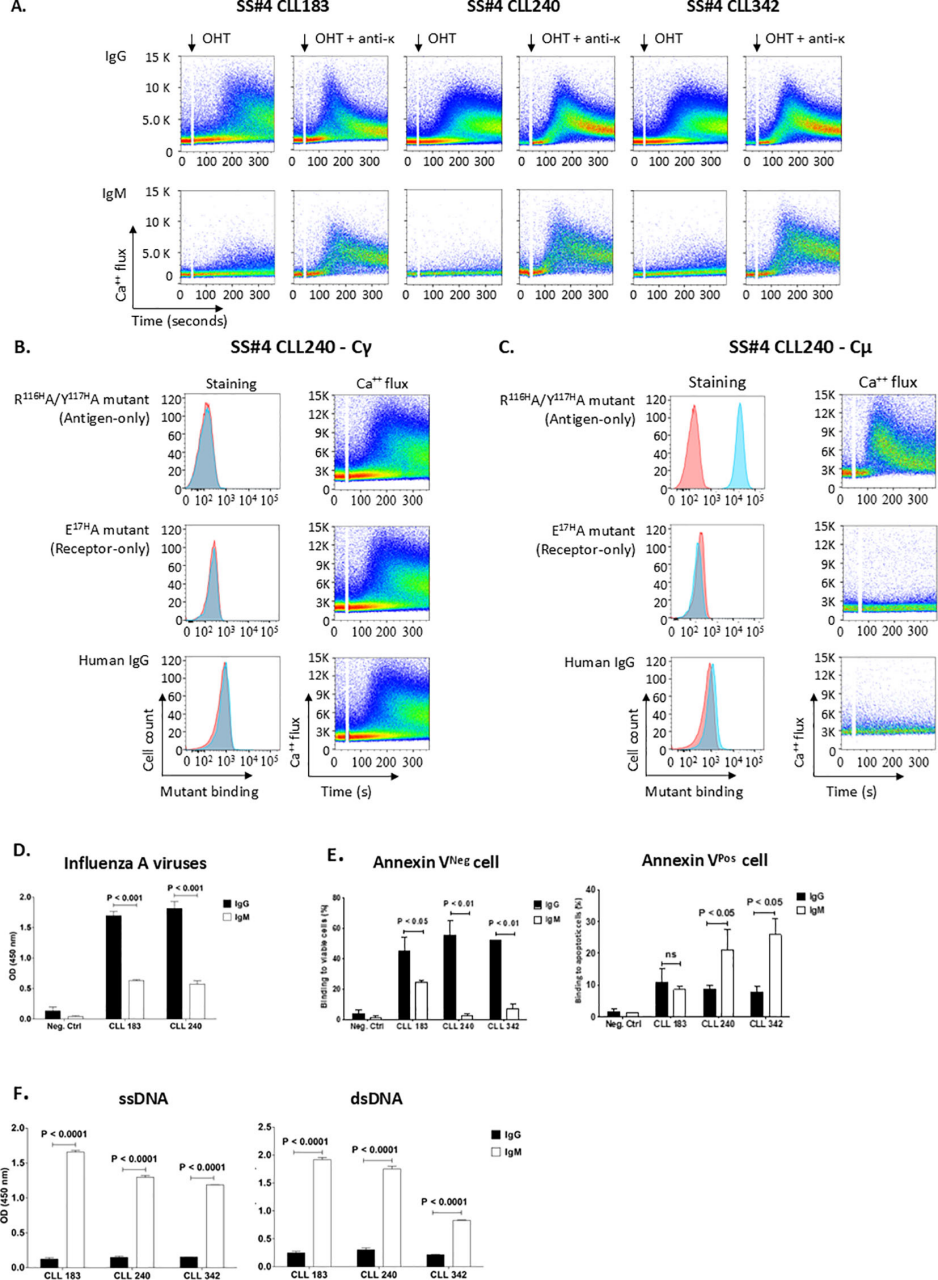
Homo-dimerization of SS#4IgGs leads to a long-lived complex with BCR signaling capacity. **(A)** Homo-dimerized SS#4IgGs induce autonomous BCR signaling. The rearranged IGHV-D-Js of wt Cγ-linked SS#4IgGs (183, 240, 342) were transfected with the appropriate IGKV-J chains into TKO cells and signaling compared to that of IGHV-D-J of Cµ-linked SS#4IGs based on Ca^++^ flux upon 4-OHT exposure. **(B)** Functional estimation of affinity of homo-dimerized SS#4IgGs. Cγ-linked 240IGs (BCR 240 Cγ) were exposed to 25μg/ml of soluble “Antigen-only” mutant R^116H^A/Y^117H^A or “Receptor-only” mutant E^17H^A that react with “Receptor IG” or “Antigen IG”. Binding to TKO cells expressing Cγ-linked mAb240 (blue) or to TKO cells transfected with empty vector (red) is compared by Ca^++^ flux in the presence of soluble mutants or human IgG upon 4-OHT exposure. **(C)** Normal human serum lacks subset #4 IG antigens. Cμ-linked SS#4IgG 240 was exposed to the soluble mutants at 25 μg/ml. Binding of these IGs to TKO cells expressing Cμ-linked IG 240 (blue) or empty vector (red) is compared. **(D–F)** The antigens ushering the transformation of normal B lymphocyte to a SS#4 leukemic clone. The Cγ regions of SS#4 IGs of CLL 183, 240 and 342 and a negative control (CLL 282) were swapped with Cμ and expressed as secreted, recombinant antibodies. Reactivity of the Cγ- and Cμ-linked IGs with influenza A virus **(D)**, viable B cell surfaces (**E**, left), apoptotic B cell surfaces (**E**, right), ssDNA (F, left) and dsDNA (F, right) was compared. Average binding of IgG vs IgM to: **(D)** Influenza A viruses 183 and 240; IgG vs IgM: 1.697 ± 0.119 OD vs. 0.630 ± 0.032 OD, *P* < 0.001; 1.816 ± 0.197 OD vs. 0.574 ± 0.093 OD, *P* < 0.001, respectively; **(E)**, left. Viable B cell surfaces, 183, 240, 342; IgG vs IgM: 44.83% ± 12.72% vs. 24.45% ± 11.36%, *P*<0.01; 55.60% ± 13.76% vs. 4.71% ± 4.89%, *P*<0.01; 51.97% ± 0.57% vs. 7.02% ± 4.76%, *P*<0.01, respectively; **(E)**, right. Apoptotic B cell surfaces. 183, 240, 342; IgG vs IgM: 10.99% ± 4.09% vs. 8.58% ± 0.86%, *P=0.256*; 8.62% ± 1.27% vs. 27.33% ± 2.37%, *P<0.01* and 7.78% ± 1.73% vs. 25.95% ± 4.87%, *p<0.05*, respectively; For both the viable and apoptotic B cell surfaces, the Cγ- and Cμ-linked negative control IG showed minimum/negligible binding (viable, IgG vs. IgM 3.95% ± 2.36% vs. 1.51% ± 0.95%, *P=0.1687*; apoptotic, 1.51% ± 1.13% vs. 1.33% ± 0.039%; *P=0.4235*). **(F)**, left. ssDNA. 183, 240 and 342; IgG vs IgM: 0.124 ± 0.023 vs. 1.657 ± 0.026, *P*<0.0001; 0.149 ± 0.017 vs. 1.296 ± 0.027, *P*<0.0001; 0.154± 0.003 vs. 1.189 ± 0.003, *P*<0.0001, respectively; **(F)**, right. dsDNA. 183, 240 and 342; IgG vs IgM: 0.251 ± 0.027 vs. 1.920 ± 0.038, *P*<0.0001; 0.303 ± 0.038 vs 1.753 ± 0.049, *P*<0.0001; 0.219 ± 0.004 vs. 0.830 ± 0.011, *P*<0.0001; respectively. Data from at least 3 independent experiments. Error bars represent SEM.

As expected (9, 17), cells expressing wt SS#4IgGs on the TKO surface membrane initiated significant Ca^++^ flux upon 4-OHT exposure (**Figure 4A**). In contrast, SS#4 expressed as IgM could not, confirming that isotype switching is necessary for self-association (**Figure 4A**). However, neither the 240 variants with intact “Antigen” nor intact “Receptor” sites could disrupt the Antigen-Receptor complex nor could they interrupt Ca^++^ flux (**Figure 4B**). Similarly, pooled, affinity-purified human IgG at concentrations of 25– 50 μg/mL (**Figure 4B**) and pooled human IGs up to 10 mg/mL (not shown) did not do so. In contrast, surface SS#4IgM, which contains intact “Receptor” but lacks “Antigen” due to absence of K241 in the human µ H chain, bound to soluble “Antigen-only” (R^116H^A/Y^117H^A) but not to “Receptor-only” variant (E^17H^A) (**Figure 4C**).

This functional estimation of “Antigen-Receptor” affinity suggests that the interaction between the two components is relatively strong and prevents disruption of the homo-dimerized IG by microenvironmental antigens. Analyses of the affinity of the two components in a soluble setting (17) corroborate our membrane estimates of the strength of the IG-IG interaction and support our conclusion. Moreover, the finding that routine screening of SS#4IgG binding reveals interactions with influenza virus HA which requires homo-dimerization supports that the self-associated state is relatively stable and long lived.

### 3.5 Tracking the influences of conventional and non-conventional antigen-binding on the transition of a normal B lymphocyte to a leukemic subset #4 IgG clone

*Influence of IG isotype*. Next, we set out to track the impact of “conventional/classical” (non-dimerized) and the “non-conventional” (homo-dimerized) antigen-binding on the development of a normal B cell bearing the SS#4IG IGHV-D-J rearrangement. Since SS#4 B cells always express IgG, we first determined if SS#4IgMs reacted with the same foreign (influenza virus HA) and self (viable B-cell membranes) antigens that the SS#4IgGs did. After creating recombinant variants of two CLL SS#4IGs (183 and 240) bearing the wt IGHV-D-J rearrangement linked to either Cµ or to Cγ and also linked with the corresponding wt IGKV-IGKJ rearrangement, binding to influenza virus and to viable and apoptotic human B cells was analyzed.

Whereas the wt Cγ-linked SS#4IGs exhibited substantial binding to influenza virus A/Texas 1/77 (H3N2) in ELISA, binding of the Cµ-linked versions of 183 and 240 IGs was significantly reduced (reduction percentage IgM vs. IgG- IG183: 62.9%, *P* < 0.001; IG240: 68.4%, *P* < 0.001; **Figure 4D**). Similar significant differences were found when we tested reactivity of the wt Cγ-linked and the Cµ-linked versions with viable human B cells by flow cytometry (**Figure 4E, left**: reduction percentage IgM vs. IgG - IG240: 91.5%, *P*<0.01; IG342: 86.5%, *P*<0.01; IG183: 45.5%, *P*<0.01; **Supplementary Figure S2A**).

Remarkably, the SS#4IgM interacted significantly with single stranded DNA (ssDNA; **Figure 4F, left**) and double stranded DNA (dsDNA; **Figure 4F, right**), whereas as expected the IgG-linked SS#4IGs did not (*P*<0.0001). Similarly, we detected binding of the SS#4IgMs, but not the SS#4IgGs, to apoptotic/dead cells (**Figure 4E, right**: **Figure 4E, right**: IG240: *P*<0.01; IG342: *P*<0.05; IG183: *P=0.256*; (**Figure 4E, right**; **Supplementary Figure S2B**).

**Figure 5:**
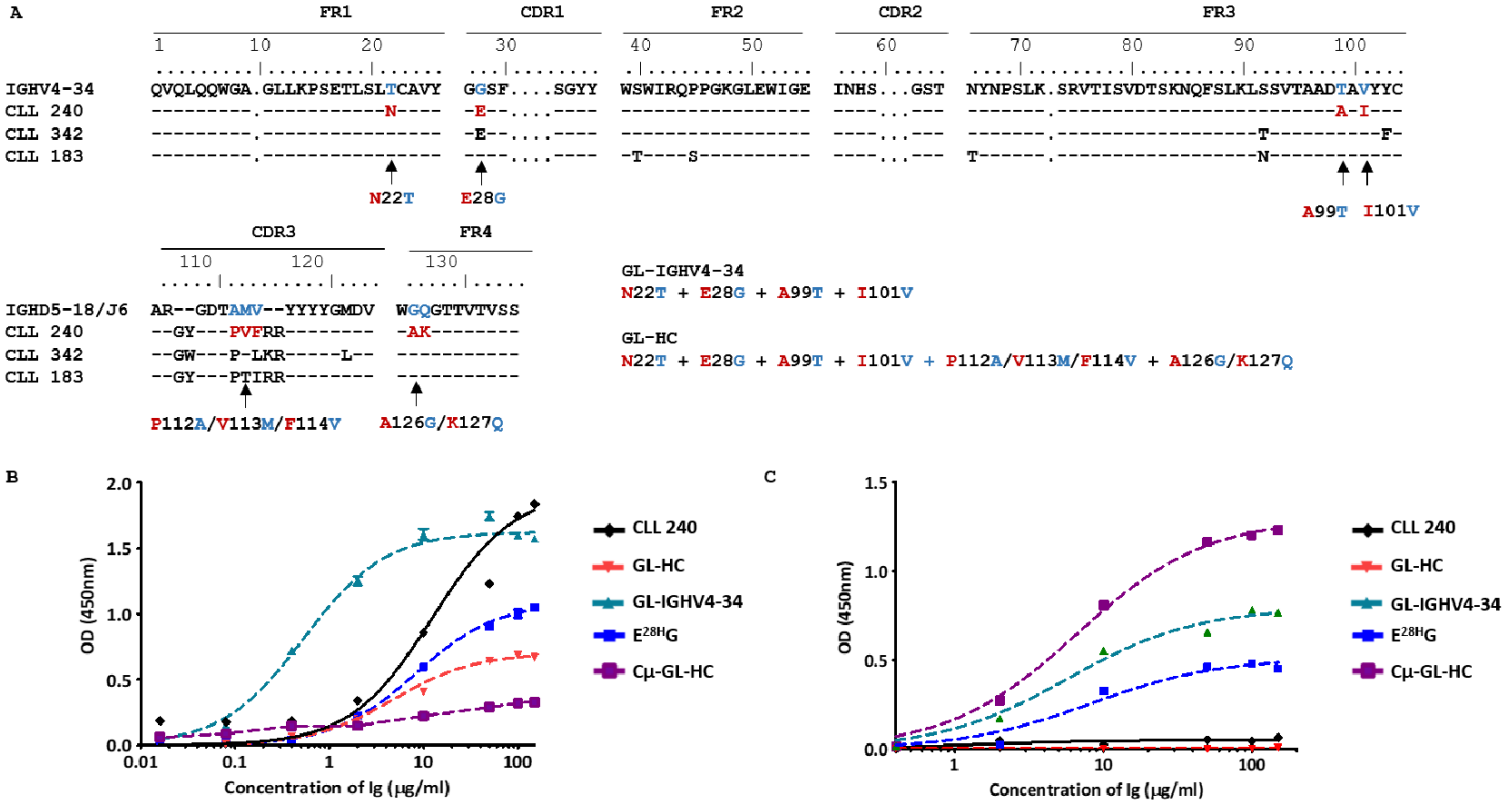
Impact of somatic mutation on the ability of SS#4IgGs to bind to influenza virus hemagglutinin and ssDNA. **(A)** The IG H variable domain of SS#4IGs (CLL 240, 342, 183) are aligned with the corresponding germline counterpart using the IMGT numbering system. A comparison of the numbering in the IMGT and that used in crystallography studies (17) can be found in **Supplementary Figure S3**. Arrows indicate the residues reverted to the germline sequence. Reactivities of the germline variants, linked to Cγ or Cμ, were tested for reactivity with **(B)** influenza virus A/Texas/1/77 (H3N2), and **(C)** ssDNA. For binding to **(B)** influenza viruses, reverting all mutations and the reversion of only E^28H^ (E28HG) led to significantly reduced binding compared wt SS#4–240 IgG (Maximum OD - wt vs. GL-HC 240: 1.838 ± 0.035 vs. 0.800 ± 0.004, *P*<0.001; wt vs. E^28H^G: 1.838 ± 0.035 vs. 1.048 ± 0.003 OD, *P*<0.001). Reverting somatic mutations in IGHV4–34 showed dramatic increase in binding affinity to influenza virus (estimated K_D_ of wt CLL240 vs. GL-IGHV4–34 are K_D_ - wt 240 vs. GL-IGHV4-34: 100.07 ± 34.54 vs. 3.63 ± 1.04 nM, *P*<0.05). For binding to **(C)** ssDNA, wt SS#4–240 IgG showed minimum signal at highest concentration. Similarly, the GL-HC revertant did not exhibit binding. The mutations in IGHV4–34 or the single revertant E^28H^G significantly increased binding to ssDNA (Maxum OD - GL-IGHV4–34 vs. WT: 0.768 ± 0.030 OD vs. 0.067 ± 0.014 OD, *P*<0.001; E^28H^G vs. WT: 0.453 ± 0.020 OD vs. 0.067 ± 0.014 OD, *P*<0.001). Cμ linked germline sequence exhibited the highest binding to ssDNA (Maxum OD – Cμ-GL-HC: 1.233± 0.027 OD)

Thus, the SS#4IgGs and their Cµ-linked variants bind distinct types of antigens, using discrete antigen binding domains. So, the emerging naïve SS#4 IgM-expressing B cell was not driven to survive and expand by antigens that drove the mature SS#4 IgG-expressing B cell. Also, these interactions were mediated by the classical/conventional/non-dimerized binding site. The antigens involved here were at least those typical autoantigens with which most CLL clones bind*Influences of somatic mutations shared by multiple SS#4IgGs on influenza virus binding, autoreactivity, and B-cell maturation*. To discriminate the effects of somatic mutations on the development and function of normal B cells bearing a SS#4 IGHV-DJ, we focused on the mutations in the IGHV-D-J rearrangement. First, we reverted all the IGHV-D-J mutations in IG240 to the germline sequence (GL-HC) (**Figure 5A**) and paired this with the wt IGKV-J. This significantly reduced reactivity with influenza virus compared to wt SS#4IgG 240 (*P*<0.001) (**Figure 5B**). Since GL-HC IgG corresponds to the IGHV-D-J germline precursor of mAb240 that was present at the time of isotype switching from an IgM^+^ to an IgG^+^ B cell, this finding indicates that somatic mutations occurring somewhere in the germline H chain variable domain enhanced binding to the virus.

Because alterations of the amino acids within the IGHV are not affected by the assembly of the IGHV-D-J and hence result from antigenic stimulation and potential selection, we next focused on somatic mutations in IGHV4-34. Specifically, all mutations in IGHV4-34 (the SS#4 shared mutation as well as the mutations unique to patient 240) were reverted to the germline sequences (GL-IGHV4-34), while retaining the remainder of the gene in the wt configuration (**Figure 5A**). Surprisingly, reverting all the somatic mutations present in IGHV4–34 led to a 28-fold increase in binding affinity compared to that of wt IG240 (*P*<0.05) (**Figure 5B**). Thus, the development of these mutations had a negative effect on HA binding and mutations developed outside of IGHV4–34 significantly enhanced binding to the virus.

To directly test the potential influence of the recurrent, stereotyped change Gly to Glu/Asp at position 28, we next solely reverted this residue to germline (E^28H^G), keeping the remainder of the molecule in the wt IgG state. Remarkably, this single reversion significantly reduced viral binding compared to wt IG240 (*P*<0.001) (**Figure 5B**). So, the acquisition of the stereotyped G28E mutation, which contributes to some extent to self-association, contributed positively to viral reactivity during B-cell development (**Figure 5B**).

Finally, we asked if the mutations tested above were involved in the lack of autoreactivity of SS#4IgGs. SS#4IgG 240 bound negligibly to DNA (**Figure 5C**). Unexpectedly, reverting the entire H chain variable domain to the germline sequence (GL-HC/wt-LC) did not lead to DNA binding. Notably, when the same germline sequence was linked to Cµ (Cμ-GL-HC), the resulting mutant showed significant binding to DNA. When only the mutations in IGHV4–34 were reverted to their germline amino acids (GL-IGHV4-34), DNA reactivity was significantly increased compared to wt SS#4IgG240 (*P*<0.001). Thus, the mutations in IGHV4-34, which reduced foreign antigen binding, also suppressed the autoreactivity of SS#4IgG. Moreover, reverting only the stereotyped, negatively charged amino acid E, which could repel DNA, back to G (E^28H^G), led to substantial increased binding to ssDNA (*P*<0.001) (**Figure 5C**).

Thus, the stereotyped G28E mutation and the mutations unique to IG240 appears to have been selected to inhibit autoantigen binding. In addition, the G28E mutation, which is shared by many SS#4 patients, increased influenza virus HA interactions. So, collectively these mutations seem to have allowed the SS#4 B cell to avoid negative selection and permit clonal survival and growth, and the stereotyped mutation could have promoted grow induced via the unconventional antigen-binding site.

### 3.6 Functional and biologic correlates of the unique structural features of SS#4IgGs

**Figure 6:**
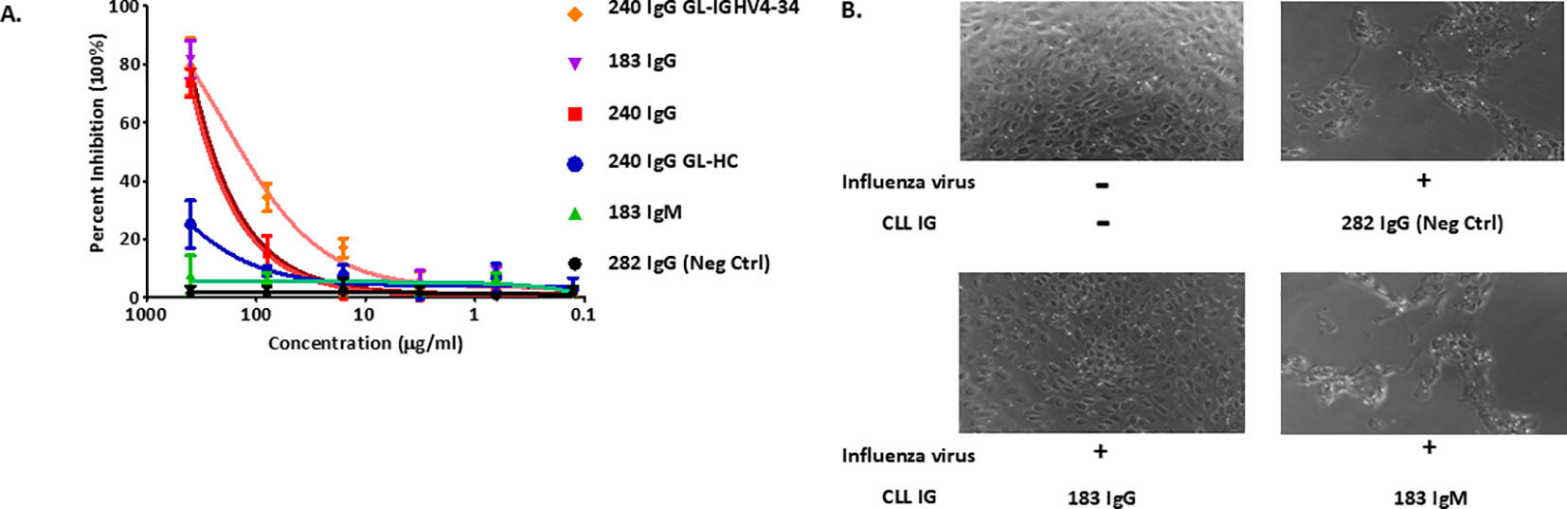
Functional and biologic correlates of the unique structural features of SS#4 IgGs and their variants. **(A)** Plaque assay. Influenza virus A/Philippines/2/82 (H3N2) was incubated with the various SS#4IGs at 37°C for 1h and then transferred to a monolayer of MDCK cells. Agarose growth media was then overlaid for 5–7 days, at which time PFU/mL were determined. **(B)** Cytopathic Effect Inhibition Assay. Cγ- and Cμ-linked SS#4IGs were incubated with influenza virus A/Philippines/2/82 (/H3N2) at a final concentration of 400μg/ml and then loaded on monolayer of MDCK cells. The morphology of host cell was observed by phase contrast microscopy. PBS buffer containing no mAbs or influenza A virus was used as non-infection reference control. CLL282, which does not react with influenza virus, was a non-neutralizing control.

In CLL, (auto)antigen binding to the BCR is critical for the survival and expansion of the leukemic clone. In the healthy state, secretion of the SS#4 IgG by a normal B cell could be of physiologic importance. Therefore, we tested if the distinct structural features of SS#4IGs might be advantageous to the host as an effector molecule. To do so, we analyzed the capacity of SS#4IgGs and its progenitors (Cµ-linked and somatic mutations reverted) to neutralize influenza virus (**Figure 6A**).

Specifically, we tested if the various IGs could protect Madin-Darby canine kidney (MDCK) cells from infection by A/Philippines/2/82 (H3N2) virus. MDCK cells, incubated with wtSS#4IGs (IgG183 and IgG240) and then exposed to the virus, were protected from influenza virus infection in a dose-dependent manner (~80% inhibition at the highest tested concentration) (**Figure 6A**). Cells incubated with the Cμ-linked SS#4 (IgM183) did not inhibit infection, even at highest concentration; this lack of reactivity was equivalent to that of the negative control. The Cγ-linked, IGHV-D-J germline precursor of mAb240 (GL-HC) showed minimal inhibition, as expected from the binding curves (**Figure 5B**). In contrast, the progenitor Cγ-linked GL-IGHV4–34 showed enhanced viral inhibition compared to wt SS#4 IG240 and was the best inhibitor among all tested variants, consistent with the ELISA binding data (**Figure 5B**).

As illustrated by phase-contrast light microscopy (**Figure 6B**), MDCK cells exposed to virus after incubation with SS#4IgG183 retained a healthy morphology and an intact, attached monolayer, resembling cells grown in the absence of virus. This differs for cells exposed to influenza virus after incubation with SS#4IgM183 in that cell numbers were remarkably reduced and the monodispersed and spherical shapes of healthy cells was lost. The latter was very similar to those incubated with the control, virus non-reactive IgG282 (**Figure 6B**).

Thus, these *in vivo* actions corroborate the *in vitro* studies above. In addition, they suggest that soluble homo-dimerized SS#4IgGs can protect against infection of cells by influenza virus. However, since the efficiency of this protection is considerably lower than that of a therapeutic mAb, the SS#4IgGs most likely function as B-cell receptors for foreign and self-antigens rather than as soluble effector molecules.

## 4 Discussion

Here, we have studied IGs from patients with CLL that are members of SS#4. These IGs differ from most other CLL antibodies in that they are always of the IgG isotype, are always somatically mutated, and react with viable (rather than apoptotic) lymphoid cell membranes. We now show four additional unique features of the SS#4IgGs.

First, in addition to binding viable lymphoid cell membranes, SS#4 IgGs also bind the foreign antigen influenza virus and its HA. Remarkably, however, this reactivity, and that to viable lymphocytes, only occurs in the homo-dimerized state. In the absence of self-association, binding to both antigens is lost, and in its place reactivity with targets typical for other CLL IGs appear, i.e., apoptotic cells and classic autoantigens such as ss- and ds-DNA. However, this autoreactivity, which is typical of other CLL IGs, is mediated by the conventional BCR of SS#4 IgGs, whereas the SS#4 unique reactivities are mediated by the non-conventional binding site acquired upon homo-dimerization.

Second, our antigen-binding and molecular modeling studies indicate that homodimerized SS#4IgGs react with shared epitopes in the stem region of the HA, consistent with their ability to appreciably bind two groups of influenza virus HAs (H3 and H1). Antibodies reacting with the stem portion of HA are less frequent in the normal B-cell repertoire and can be broadly neutralizing (58–60). Because we could not disrupt IG-IG self-association by incubation with high amounts of soluble SS#4 components or IGs in normal serum, the non-conventional antigen binding site is relatively long-lived *in vivo*. These findings, along with the fact that reactivity of SS#4IgGs with autoantigens and apoptotic cells is only seen when eliminating homo-dimerization, suggest that reactivity with influenza virus HA and with viable lymphoid cell surfaces occurs in patients. Although exposure to influenza is periodic, since the SS#4IgGs bind to the stem region of multiple HAs, yearly exposure is feasible. However, more relevant biologically might be the reactivity with viable lymphocyte cell surfaces since these interactions would occur continuously in *in vivo*.

Third, our modeling studies suggest that the interaction of the self-associated SS#4IgG with influenza virus HA involves portions of the homodimerized IG molecule that are mostly outside of the areas used for classical BCR binding. Specifically, for the Antigen IG portion of the Antigen-Receptor complex, there is a large interface with HA that consists of the constant region of the IG H chain, involving 23 amino acids; no residues in the variable domains of the H or L chains of the Antigen IG interact with the HA. Similarly, for the Receptor IG part of the complex, 9 residues in the FRs of the variable domain of the H chain and 2 residues in the FR of the L chain engage HA; only 5 residues in the VH CDR1 and 2 in the VL CDR2 of the Receptor IG interact with the virus HA. To the best of our knowledge, this is the first demonstration of antigen-binding that requires a conformational structure acquired by homodimerization of an IG molecule and that involves primarily constant and framework region residues. Since the identity of the epitope on the surface of viable lymphoid cell surfaces has yet to be defined, we could not perform modeling studies for this target.

Lastly, these studies have identified key residues that support reactivity with influenza virus HA and viable B-cell surfaces that appear to also be relevant to homo-dimerization. For example, self-association requires the presence of a Lys at position 241 (IMGT numbering. **Supplementary Figure S3**) of the H chain of the Receptor IgG. Consistent with this, we have here shown that the absence of this amino acid, either because the requisite Cγ chain has been replaced by a Cµ chain which does not have a Lys at this site, or because the Lys is changed to another residue in the CH γ chain, eliminates autonomous signaling, which is a consequence of homo-dimerization, as well as preventing (auto)antigen binding. Thus, when SS#4 IgGs are self-associated, the Lys at 241 of the Antigen IG, is occupied. However, intriguingly, the available Lys at 241 on the Receptor IG interacts with the influenza virus HA. Notably, this residue appears to have two functions in SS#4 IgGs: promoting homo-dimerization and binding HA, albeit on different components of the IG-IG complex.

Additionally, SS#4 IgGs from multiple patients display an E^28H^G somatic mutation in the FR1 of the IGHV4–34 gene at position 28 (IMGT numbering). The crystal structure of the homo-dimerized SS#4 suggests that this stereotyped mutation is proximal to the interface of the complementary IG, albeit not directly in contact with the opposite molecule, and could allow for charge-mediated interactions and contribute to IG-IG contact. Notably, this stereotyped mutation significantly enhances interactions with influenza virus HA.

Using this information, we have tried to recapitulate the development of a SS#4IgG leukemic cells from a naïve B lymphocyte, focusing on the binding of foreign and auto- antigens by the SS#4 conventional and non-conventional binding sites. Switching the SS#4IgG back to its initial developmental IgM state, which eliminates self-association, and reverting all the somatic mutations in the IGHV-D-J to their germline sequences leads to an IG that reacts significantly with apoptotic cells and ss- and ds-DNA; both typical for most CLL IGs. Thus, this B cell will be driven at least in part by autoantigens bound by the conventional BCR. However, because of the inability to homo-dimerize, the IgM-expressing, normal precursor will not benefit from autonomous signaling.

Isotype class switching to IgG without IGHV mutations, a temporal sequence consistent with other studies (61), allows homo-dimerization, the development of autonomous signaling, and the acquisition of the ability to bind to influenza virus HA and viable B cells. In fact, switching to IgG without developing IGHV somatic mutations leads to the greatest level of influenza virus binding via the non-conventional binding site. However, although isotype switching eliminates reactivity with apoptotic cells, it results in significant interactions with DNA. Notably, acquisition of the stereotyped mutation at position 28 diminishes this autoreactivity, while enhancing influenza virus HA binding. Thus, the repetitive presence of this mutations among multiple SS#4 patients indicates selection for two functions: binding to HA and inhibiting binding to DNA. The latter allows the clone to avoid tolerance mechanisms and survive and grow, being aided by ongoing autonomous BCR signaling.

The homo-dimerized SS#4IgG prevents influenza virus infection of target cells *in vitro*, albeit at an efficiency much less than clinically beneficial therapeutic IGs. Since in CLL the level of secreted IGs is minimal, it is unlikely that patients with CLL would have sufficient amounts of soluble SS#4IgGs to protect from viral infection. However, the SS#4IgG clone might benefit from the presence of non-conventional binding to influenza virus and HA, since on the cell membrane the non-conventional SS#4IgG receptor would be multimeric, fostering higher affinity for the viral HA and viable lymphoid cell membranes. In addition, the target HA is displayed as multiple copies on infected cells, again enhancing non-conventional receptor – target binding efficiency. Thus, SS#4IgG cells could receive dual stimuli via the BCR: a chronic stimulus delivered by self-association via the conventional BCR and stimulation delivered by viral antigen and viable lymphocyte encounter.

This scenario raises the possibility that, if a patient with a SS#4IgG clone becomes infected with influenza virus or is inoculated with anti-influenza vaccine, the SS#4IgG CLL cell could receive survival and growth signals, leading to disease progression. In this regard, it is possible that the high affinity of homo-dimerization would lead to ongoing autonomous signaling through the conventional BCR, leading to desensitization and anergy. The latter appears to be the case *in vitro* as SS#4IgG cells are unresponsive to external stimuli. The next consideration is how will intermittent signaling from influenza infection and immunization or frequent signaling by interacting with viable B cells (leukemic and normal) affect the SS#4IgG clone *in vivo?* Will these enhance desensitization and anergy or would the latter override the former and lead to clonal expansion? In this regard, interactions of the SS#4IgG clone with live leukemic B cells, especially in tissues, are likely to result in ongoing signaling via the unconventional binding site.

Finally, it will be interesting to see if the creation of a non-conventional binding site on B cells based on homo-dimerization (or other self-interactions) is unique to this subset of patients with CLL or if the self-association principle is operable in other normal and abnormal settings. Since homo-dimerization is a feature of most/all CLL IGs and of the IGs of certain lymphomas, this phenomenon may not be idiosyncratic to SS#4IGs in B-cell leukemias/lymphomas.

## Data Availability

Modeling can be viewed at: https://figshare.com/articles/figure/Structure/28239857?file=51805436. 373 374 375.
