# Supplementary material for: Conventional and non-conventional antigen-binding sites promote the development and function of chronic lymphocytic leukemia stereotyped subset #4 clones
Source: Front Immunol. 2025 Aug 21;16:1607189. doi: 10.3389/fimmu.2025.1607189 (PMC12408587; doi:10.3389/fimmu.2025.1607189)
Supplement: Supplementary file 1 [file DataSheet1.pdf]

Supplemental Information for:

Conventional and non-conventional antigen-binding sites promote the development and function of chronic lymphocytic leukemia stereotyped subset #4 clones

Yun Liu[1,2], Dzmitry Padhorny[3,4], Rosa Catera[1,2], Antonella Nicolo[5], Xiao-Jie Yan[1,2], Stan Xiaogang Li[3,4], Anastasia Iatrou[6], Steven L. Allen[1,2], Jonathan E. Kolitz[1,2], Kanti R. Rai[1,2], Massimo Degano[7,8], Paolo P. Ghia[9,10], Charles C. Chu[1,2,*], Florian Krammer[11,12], Hassan Jumaa[5], Kostas Stamatopoulos[6], Dmytro Kozakov[3,4], Nicholas Chiorazzi[1,2]

[1] Northwell, New Hyde Park, NY, USA
[2] The Feinstein Institutes for Medical Research, Manhasset, NY, USA
[3] Department of Applied Mathematics and Statistics, Stony Brook University, Stony Brook, NY, USA
[4] Laufer Center for Physical and Quantitative Biology, Stony Brook University, Stony Brook, NY, USA
[5] Institute of Immunology, Ulm University Medical Center, Ulm, Germany
[6] Institute of Applied Biosciences, Centre for Research and Technology Hellas, Thessaloniki, Greece
[7] Università Vita-Salute San Raffaele, Milano, Italy
[8] Division of Immunology, Transplantation, and Infectious Diseases, IRCCS Scientific Institute San Raffaele, Milano, Italy
[9] Medical School, Università Vita-Salute San Raffaele, Milano, Italy
[10] B Cell Neoplasia Unit and Strategic Research Program on CLL; IRCCS Ospedale San Raffaele, Milano, Italy
[11] Department of Microbiology, Icahn School of Medicine at Mount Sinai, New York, NY, USA
[12] Ignaz Semmelweis Institute, Interuniversity Institute for Infection Research, Medical University of Vienna, Austria
* Current address: Department of Medicine, Wilmot Cancer Institute, University of Rochester Medical Center, Rochester, NY, USA

Supplemental Materials and Methods.

Antibody specificity profiling. Purified CLL IGs were tested by the vendor for binding to the antigens displayed on ProtoArray® Human Protein Microarray V 4.0 (Invitrogen) was carried. Non-specific binding was reduced by incubation with blocking buffer (100 mM Sodium Phosphate, pH 7.4, 200 mM NaCl, 0.08% Triton X100, 25% glycerol, 20 mM reduced glutathione, 1.0 mM DTT, 1% Hammarsten Grade casein) with gentle agitation in QuadriPERM 4-well trays (Greiner) for 1 hour at 4°C. Various antibodies were diluted in PBST buffer (1X PBS, 0.1% Tween 20, 1% Hammersten

Grade casein) to a concentration of 10 and 50 μg/ml and added to arrays under a Lifterslip™ (Erie Scientific). Arrays were incubated at 4°C for 20 hours in a humidity chamber, transferred to a 4-well tray, and washed 5 times (5 minutes per wash) at 4°C with gentle agitation in 5 ml PBST buffer. Each array was then incubated with Alexa Fluor®647-conjugated goat anti-human IgG antibody (1 μg/ml) with gentle shaking in 4well trays at 4°C for 90 minutes. Subsequently, the secondary antibody was removed, and the arrays washed as described above and quickly rinsed in water. Arrays were dried by spinning in a tabletop centrifuge equipped with a plate rotor at 1000 rpm for 2 minutes. Arrays were then scanned using an Axon GenePix 4000B fluorescent microarray scanner.

Autonomous cell signaling measured by Ca^{++} flux. To measure intracellular calcium release upon receptor stimulation, ~ 10^6 cells were transferred into a FACS tube and centrifuged for 5 minutes at 1200 rpm at 4°C. Meanwhile, staining solution was prepared by adding 25 µl DMSO, 113 µl FBS and 25 µl Pluronic® F-127 to tube containing 50 µg lyophilized Indo-1 AM dye, a cell-permeant and UV-excitable radiometric Ca^{++} indicator which exhibits a shift in emission upon Ca^{++} releasing into the intracellular space. The mixture was incubated for 5 min at RT and in the dark. After removal of the supernatant from the centrifuged cells, the pellet was resuspended in 1 ml Iscove's media supplemented with 1% FCS (Iscove's 1% FCS) and stained with 15 µl of the Indo-1 AM mixture. Cells were later incubated in the dark for 45 min at 37°C (in the cell incubator), and tubes were flipped every 15 minutes. At the end of the incubation period, cells were spun down for 5 min at 1200 rpm at 4°C. Later cells were washed with 1 ml Iscove's 1% FCS, and the pellet was resuspended in 500 µl of Iscoves medium 1% FCS. To avoid bleaching, tubes were kept on ice and in the dark until measurements were performed using a BD LSR Fortessa instrument equipped with a 355nm UV laser. Immediately before measurement, cells were warmed in a 37°C water bath for ~10 minutes to reach their physiological temperature. The calcium baseline was recorded for 40 seconds before adding 4-hydroxytamoxifen (OHT 2µM final, for TKO cell analysis). For cross-linking Igκ, polyclonal mouse (Southern Biotech) anti-Igκ (10 µg/ml final concentration), the mixture was immediately added to cells after the last wash step.

Table S1. Characteristics of Chronic Lymphocytic Leukemia B-Cell Receptors That Were Tested in ProtoArray® Human Protein Microarrays

CLL ID	Subset	IGHV	IGHD	IGHJ	HCDR3	IGK/LV	IGK/LJ	K/LCDR3	Mutation Status IGHV (%)
154	1	IGHV1-18	IGHD6-19	IGHJ4	CAREQWLVLSHFDYW	IGKV1-39/1D-39	IGKJ1	CQQSYSTPPWTF	2.4%
270	1	IGHV1-2	IGHD5-12	IGHJ4	CARVQWLGLRHFDYW	IGKV1-39/1D-39	IGKJ2	CQQSYSTPPYTF	0.0%
340	1	IGHV1-2	IGHD6-19	IGHJ4	CAREQWLVLKNFDYW	IGKV1-39/1D-39	IGKJ2	CQQSYSTPPYTF	0.0%
360	1	IGHV1-3	IGHD6-19	IGHJ4	CAREQWLVLNYFDYW	IGKV1-39/1D-39	IGKJ2	CQQSYSTPPYTF	0.0%
282	2	IGHV3-21	IGHD1-26	IGHJ6	CARDANGMDVW	IGLV3-21	IGLJ3	CQVWDSSSDHPWVF	2.4%
412	2	IGHV3-21	NA	IGHJ6	CARDQNGMDVW	IGLV3-21	IGLJ3	CQVWDSSSDHPWVF	2.0%
183	4	IGHV4-34	IGHD5-18	IGHJ6	CARGYGDTPTIRRYYYYGMDVW	IGKV2-30	IGKJ2	CMQGTHWPPYTF	3.1%
240	4	IGHV4-34	IGHD3-10	IGHJ6	CARGYADTPVFRRYYYYGMDVW	IGKV2-30	IGKJ2	CMQGTHWPPYTF	3.1%
342	4	IGHV4-34	IGHD5-18	IGHJ6	CARGWGDTPMLKRYYYYGLDVW	IGKV2-30	IGKJ1	CMQGTHWPWTF	2.8%
68	6	IGHV1-69	IGHD3-16	IGHJ3	CARGGDYDYVWGSYRSNDAFDIW	IGKV3-20	IGKJ4	CQQYGSSPTF	0.0%
258	6	IGHV1-69	IGHD3-16	IGHJ3	CARGGIYDYVWGSYRPNDAFDIW	IGKV3-20	IGKJ1	CQQYGSSPGTF	0.0%
246	7A	IGHV1-69	IGHD3-3	IGHJ6	CARSDQNYDFWSGYFRYYGMDVW	IGKV3-20	IGKJ1	QQYGSSPET	0.3%
355	7B	IGHV1-69	IGHD3-3	IGHJ6	CARADLPYYDFWSGMYYYGMDVW	IGKV1-5	IGKJ1	CQQYNSYQTF	0.0%
114	8	IGHV4-39	IGHD6-13	IGHJ5	CARRFGYSSSWYGLDWFDPW	IGKV1-39/1D-39	IGKJ1	CQQSYSTPRTF	0.0%
657	8	IGHV4-39	IGHD6-13	IGHJ5	CASKTGYSSSWYGRDWFDPW	IGKV1-39/1D-39	IGKJ1	CQQSYSTPRTF	0.3%
845	8	IGHV4-39	IGHD6-13	IGHJ5	CASSTGYSSSWYSPTNWFDPW	IGKV4-1	IGKJ4	CQQYYTTPPTF	0.0%
366	22	IGHV3-21	IGHD3-3	IGHJ6	CARGVLNYDFWSVYYYYGMDVW	IGKV4-1	IGKJ4	CQQYYSTPLTF	0.0%
D013	28	IGHV1-2							
14	31	IGHV1-69	IGHD3-3	IGHJ6	CATKNDFWSGYYEGYYYYYYMDVW	IGLV3-1	IGLJ1	CQAWDSSTCYVF	0.0%
415	NA	IGHV1-3	IGHD3-10	IGHJ4	CARRPESGYSFVTPFDYW	IGKV1-39/1D-39	IGKJ2	CQQSYSTPPHTF	0.0%
376	NA	IGHV1-24	IGHD4-17	IGHJ1	CATSAFTVTHAEYFQHW	IGKV3-11	IGKJ1	CQQRSNWPWTF	0.0%
358	NA	IGHV1-69	IGHD1-26	IGHJ5	CAVLPSPLVGATQIWGDYW	IGKV3-20	IGKJ1	CQQYGSSPPTF	6.9%
562	NA	IGHV3-21	IGHD6-19	IGHJ5	CVRDEITVAATRPCPW	IGLV3-21	IGLJ3	CQVWDSSDDHPWVF	1.7%
169	NA	IGHV3-33	IGHD3-10	IGHJ4	CAREGGVTGQGGFDYW	IGLV1-44	IGLJ3	CAAWDDSLNGWVF	8.8%
260	NA	IGHV4-38-2	IGHD2-2	IGHJ6	CARAEIVVVPAAYYYYYGMDVW	IGKV1-27	IGKJ3	QKYNSAPQVT	0.0%
DO8	NA	IGHV4-34	IGHD6-13	IGHJ4	CARGGRAAAGKGLLDYW				
141	NA	IGHV4-34	IGHD2-2	IGHJ5	CARGDWRIVVVPAAVDTAMAANWFDPW	IGKV1-27	IGKJ2	CQKYNSAPRMYTF	0.0%
947	NA	IGHV4-34	IGHD3-22	IGHJ4	CARTNHYDSSGYYLPYW	IGKV3-20	IGKJ1	CQHYDTSPRTF	5.6%
255	NA	IGHV4-59	IGHD3-22	IGHJ4	CARHRGYESSGYYSSYFDYW	IGLVL3-1	IGLJ2	CQAWDSSTVVF	4.2%

Table S1. Characteristics of CLL IGs tested in ProtoArray® Human Protein Microarrays. Subset, IGHV-D-J and IGK/LV-J rearrangements, and amino acid sequences of CDR3s of CLL IGs are provided. Also shown is the mutation status of the IGHV gene for each CLL IG. "NA" indicates the IG does not belong to a stereotyped subset. CLL 947 was not tested in ProtoArray® Human Protein Microarrays but is included because it was tested in the confirmation ELISAs.

Figure S1.

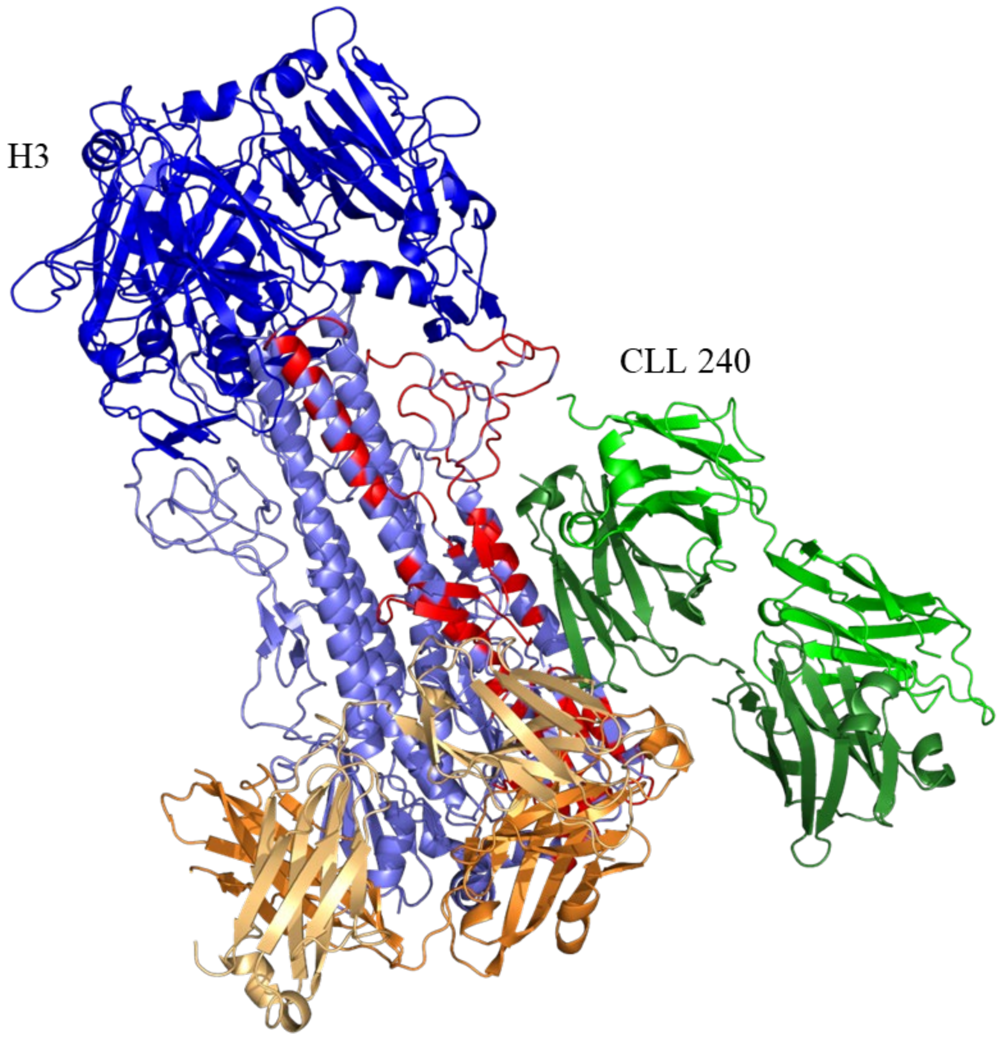

Figure S1. Difference in binding of H3 vs H2 to SS#4IgG. Residues differing between H3 and H2 stem region are highlighted in red. *Blue: hemagglutinin; orange: "receptor" antibody; green: "antigen" antibody*.

Figure S2.

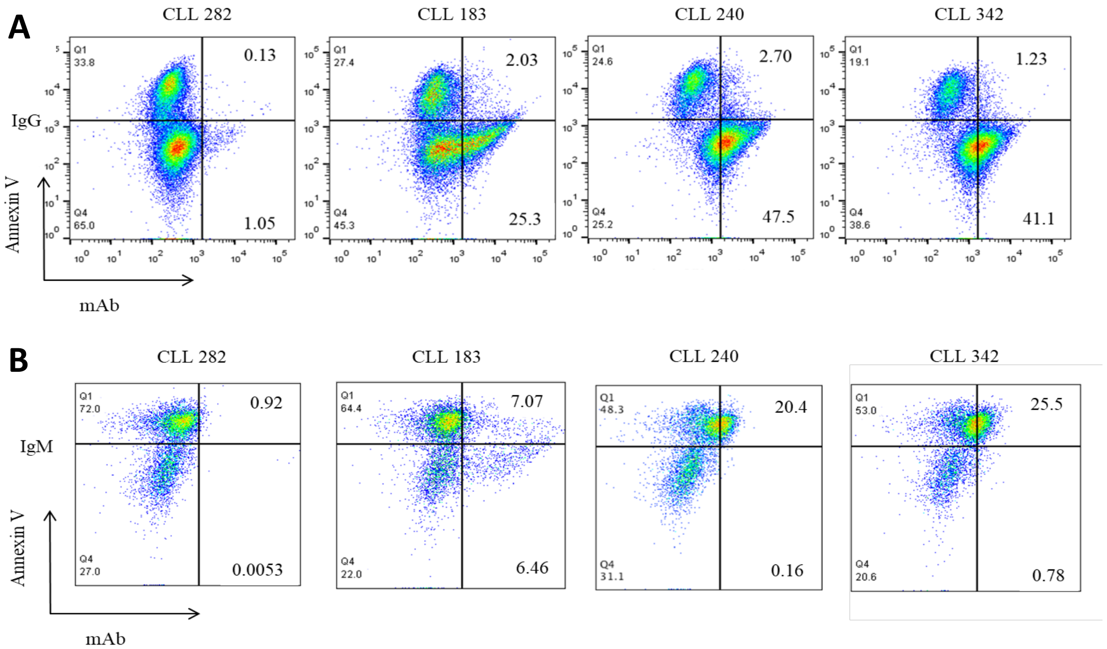

Figure S2. Cγ (A) and Cμ (B) linked SS#4IGs exhibit distinct reactivities to human B lymphocytes. Three SS#4IGs (CLL183, 240, 342) are compared with mAb282. All IGs were linked with the γ or μ IG H chain for comparison. IGs were incubated with Ramos B-cell line cells under nonpermeabilizing conditions. Apoptosis was measured by annexin V staining. IGs were biotinylated and detected by FITC-streptavidin.

```
                 FR1            CDR1          FR2           CDR2                          FR3
                 ____________________________________________________________________________________________________________
                 1        10        20        30        40        50        60        70        80        90        100
                 .........|.........|.........|.........|.........|.........|.........|.........|.........|.........|....
IMGT             QVQLQQWGA.GLLKPSETLSLNCAVYGESF....SGYYWSWIRQPPGKGLEWIGEINHS...GSTNYNPSLK.SRVTISVDTSKNQFSLKLSSVTAADAAIYYC
Minici et al     QVQLQQWGAGLLKPSETLSLNCAVYGESFSGYYWSWIRQPPGKGLEWIGEINHSGSTNYNPSLKSRVTISVDTSKNQFSLKLSSVTAADAAIYYCARGYGDTPV


                    CDR3            FR4                                                          CH1
                 ____________________________________________________________________________________________________________
                     110       120       130       140       150       160       170       180       190       200
                 .....|.........|.........|.........|.........|.........|.........|.........|.........|.........|........
IMGT             ARGYGDTPVFRRYYYYGMDVWAKGTTVTVSSASTKGPSVFPLAPSSKSTS...GGTAALGCLVKDYFP..EPVTVSWNSGALTS....GVHTFPAVLQSS....
Minici et al     FRRYYYYGMDVWAKGTTVTVSSASTKGPSVFPLAPSSKSTSGGTAALGCLVKDYFPEPVTVSWNSGALTSGVHTFPAVLQSSGLYSLSSVVTVPSSSLGTQTYI

                              CH1                                H
                 ___________________________________________________
                 210       220       230       240       250
                 .|.........|.........|.........|.........|.......
IMGT             ..GLYSLSSVVTVPSSSL...GTQTYICNVNHKP..SNTKVDKRVEPKS
Minici et al     CNVNHKPSNTKVDKRVEPKS
```

Figure S3. Numbering alignment of IMGT (international **I**m**M**uno**G**ene**T**ics information system**) and crystallization study** (Minici et al. 2017; ref #17 in main text) **of the CLL 240 BCR Fab fragment sequence**. **(A) Heavy Chain**, including IGHV4-34/D5-18/JH6, CH1, and partial hinge (H) region. **(B) Light Chain**, including IGKV2-30/JK4 and complete constant region. Framework and Hinge regions are indicated by shade. Residues mentioned in both numbering systems are in Red.
